# Low-intensity Electrical Stimulation in Wound Healing: Review of the Efficacy of Externally Applied Currents Resembling the Current of Injury

**Published:** 2008-05-16

**Authors:** Konstantine C. Balakatounis, Antonios G. Angoules

**Affiliations:** School of Health and Social Care, Oxford Brookes University, England, United Kingdom;; Filoktitis Medical Center (Center of Excellence in Physical Medicine and Rehabilitation), Athens, Greece; and; Academic Unit of Orthopaedic Surgery and Trauma, Leeds Teaching Hospitals, Leeds, England, United Kingdom

## Abstract

**Objective:** Low-intensity currents (LIC) have gained popularity during the last years, and nowadays the majority of electrotherapy units may produce LIC. On wounding, the body produces a current, the current of injury, which promotes healing. Still, this current may gradually decrease resulting occasionally to delayed or limited wound healing. Thus, by applying the same LIC externally, healing may be accelerated by sustaining the LIC throughout the healing phases. The first review of research studies on the effect of LIC on wound healing is attempted, which can be considered useful for the practicing clinician, to provide an overview of current evidence on the effectiveness of LIC and provide protocols of treatment. **Methods:** Comprehensive review of randomized-controlled trials investigating the effect of LIC on wound healing. **Results:** The review revealed that LIC promote wound healing and appear to be effective in the range of 200–800 μA. The direct current may be continuous or pulsed and polarity may or may not be reversed. **Conclusion:** Research available indicates that LIC accelerate wound healing. Further research is required to clarify the healing effects of LIC on wounds.

In 2002, electrical stimulation was approved in the United States, for Medicare coverage for the treatment of nonresponding to standard wound-healing strategies, pressure, diabetic, stasis, and arterial ulcers. The approval of electrical stimulation constitutes an indication of the growing acceptance and evidence for its application for wound healing.^1^

Electrical stimulation may be highly variable in form and parameters. There is a substantial number of research studies on wound healing,^2^ but there is rarely a differentiation among types of electrical current. Still, various forms of currents such as direct currents, pulsed direct currents, high- or low-voltage pulsed currents, alternative currents, and low-intensity currents (LIC) are available. Therefore, the identification of effective forms/parameters of currents, which are supported by randomized-controlled trials, may be considered as clinically relevant.

A review of the effectiveness of LIC on wound healing was considered important by the authors, because LIC resemble the currents produced by the human body on wounding; therefore, there is a reasonable question whether this particular form of currents may be a beneficial range of amplitude for wound healing despite the very low amplitude.

The purpose of the study was to concentrate available research on LIC stimulation for wound healing and conduct the first review on the specific topic, to investigate its effectiveness, guide clinicians by providing treatment protocols, and stimulate further research on LIC and wound healing.

## DEFINITION AND TECHNICAL SPECIFICATIONS

Low-intensity electric currents or microcurrents (MCs) are currents of an intensity less than or equal to 1 mA (1000 μA, μA = microampere). The current may be direct or alternating of varying—mainly rectangular—waveforms, frequency, and pulse duration. Low-intensity currents were formerly known as MC electrical neuromuscular stimulators, but were later named microcurrent electrical stimulator (MES) (MC electrical stimulator).^2^

Microcurrents are produced by low-voltage generators or combined electrotherapy units. Such generators or units can produce a range of waveforms, from monophasic to square or rectangular biphasic, with a range of frequencies from 0.3 to 50 Hz. Electrotherapeutic units of low voltage may produce currents of intensities up to a few milliamperes in which case sensory stimulation or muscular stimulation results. Pulse duration may also be modified from 1 to 500 milliseconds at low frequencies or may be preselected when pulsed current is utilized.^2^

## CURRENT OF INJURY

In 1843, Dubois-Reymond reported a current of an intensity of 1-mA exiting human skin wounds. It was later confirmed that wounds create a surrounding electric field, the “current of injury,” which was found to be of an intensity less than 1 mA.^3^,^4^ The current of injury extends up to a radius of 2–3 mm around the wound, and the gradient gradually decreases from 140 mV/mm to 0 mV/mm.^5^,^6^ It also appears that the transportation of Na^+^ into the cell, through the cell membrane, maintains skin “battery” of a potential difference of 20 to 40 mV, the negative pole being outside the cell.

It has been supported that the current of injury can be maintained if a moist occlusive dressing is applied, but will gradually decrease if the wound is left open and unprotected.^6^,^7^ As the wound heals, the current of injury is also reduced.^6^

By considering that healing appears to be promoted through the use of occlusive dressings,^8^,^9^ which retain the current of injury within the wound environment, it can be and has been postulated^10^ that the current of injury plays a significant role in wound healing.^11^ Therefore, it can be claimed that LIC may resemble the natural electric field/current created following injury, thus enhancing a complex biological mechanism of wound healing.^12^ One of those mechanisms is galvanotaxis. *Galvanotaxis* can be defined as the directional migration of various types of cells,^13^,^14^ such as endothelial cells,^15^ and keratinocytes, thus enhancing reepitheliazation.^16^,^17^ The biological processes underlying galvanotaxis are under investigation, 1 proposed mechanism being lateral electrophoresis resulting in changes in the plasma membrane and possibly affecting protein redistribution.^11^

## METHODOLOGY

Initially, 4 electronic databases (MEDLINE, CINAHL, EMBASE, and PeDRO) were searched for clinical studies from 1966, or earliest year available on the database to March 2008.

An attempt to identify studies using LIC wound healing was made through the implementation of a search strategy. A combination of the following key words was employed: “low-intensity current,” “low-intensity stimulation,” and “microcurrents” in combination with the key words “wound healing,” “ulcer,” and “ulceration.” References in articles were scanned for additional clinical studies. By scanning references in the retrieved articles, it became clear that in numerous studies, MCs (LIC) were used in treatment but were not defined as such. Instead, they were referred to as “electrical stimulation,” a term that includes LIC as well. This fact led to a new broader search strategy using electrical stimulation as a key word instead of the terms MCs or low-intensity stimulation. Therefore, the key word electric stimulation was also used.

All results from the searches were carefully scanned for studies related to LIC and wound healing.

The final search strategy was conducted by 2 reviewers independently, and final studies retrieved and included in the study (*n* = 4) were reproduced successfully by a medical doctor and physical therapist not involved in the study.

The inclusion criteria consisted of clinical trials investigating healing of noninfected wounds in human subjects. No restriction on age and date of publication was applied.

Exclusion criteria were as follows:

- studies investigating healing of infected wounds;
- studies in languages other than English, German, French, Spanish, and Greek; and
- high-voltage currents and all other currents other than LIC.

## EFFECTIVENESS OF DIFFERENT TYPES OF LIC

The efficacy of LIC on wound healing has been investigated by several clinical studies on human subjects.

### Low-intensity direct current

Low-intensity direct current (LIDC) is the most common type of LIC studied in research. Wolcott et al^18^ studied wound healing resulting from application of LIDC in 83 patients with ischemic wounds. Three sessions per day took place, each lasting 2 hours. Intensity ranged from 200 to 800 μA, the negative electrode was placed on the wound and the positive electrode proximally. After 3 days, polarity was reversed provided that no infection had appeared. In the event of presence of infection, reversal was postponed until infection had subsided and was then delayed for an additional 3 days. Afterward, polarity was reversed each time healing reached a plateau. The rationale of the delay of polarity reversal may be attributed to the study of Rowley et al,^19^ where by placing the negative electrode on the wound in similar parameters, the current presented with antimicrobial effects. Forty-five percent of wounds healed completely around a mean of 9.6 weeks, and the rest reached partial healing up to 64.7% over 7.2 weeks. Direct comparison of 2 treatments, standard treatment versus LIC, on the same subjects also took place, a fact that eliminated confounding factors stemming from differences among individuals such as age, sex, general health, and underlying pathology (eg, diabetes). Eight of the patients presented with bilateral wounds. One side was treated with LIDC (*n* = 8) and the other received standard care (*n* = 8). Six of 8 LIDC-treated ulcers, completely healed, while the rest 2 of 8 healed up to 70%. In the other side, 3 of 8 ulcers did not heal, 3 of 8 healed less than 50%, and 2 out of 8 healed no more than 75%. In another clinical study,^20^ LIDC stimulation was applied to 6 patients with bilateral ischemic skin ulcers. The parameters of LIDC were same as in the study by Wolcott et al,^18^ only polarity was reversed once. One side received standard treatment, whereas the other side ulcer received the same treatment plus LIDC stimulation. The healing rate of the non-LIDC side was 14.7% compared with 30% in the LIDC-treated side. A significant enhancement of healing was observed. A total of 100 patients also received LIDC treatment on ischemic wounds including the six patients previously mentioned. Mean healing rate amounted to 28.4% per week.

The positive effect of LIDC on chronic leg ulcers nonresponsive to other treatment has also been supported in a case study by Assimacopoulos et al,^21^ in which, LIDC was applied on 3 patients with venous leg ulcers. Healing occurred in all 3 patients in 6 weeks, by applying a current of 100 μA. No control group was available, and being a case study, the strength of the results is somewhat limited.

Carley and Wainapel^22^ applied LIDC (200–800 μA) on 30 patients with ulcers of various pathologies located over the sacrum or the lower limb below the knee. Patients were assigned in an electrical stimulation treatment group (*n* = 15) or conventional treatment group (*n* = 15) matched according to age, diagnosis, etiology, and wound size, thus ensuring that confounding factors were controlled to a considerable extent. Both groups received standard conservative treatment. The treatment group received additional electrical stimulation of 200 to 800 μA for 2 hours, twice daily, with an interval of at least 2 to 4 hours, 5 days per week, for 5 weeks. The negative electrode was placed on the wound and the positive electrode proximally. Reversal of polarity took place, as in the study by Wolcott et al,^18^ and treatment was continued until full wound healing was reached.

Results demonstrated statistically significant acceleration of wound healing of 1.5 to 2.5 times greater in the LIC group with respect to the conventional treatment group, and furthermore, less debridement was required, as well as less discomfort and resilient scars were observed. Healing was therefore enhanced by LIC stimulation (Table 1).

### Low-intensity pulsed direct current

Low-intensity current provides minor stimulation to the healing site, being an LIC. One might expect that by using a pulsed form of this current, effectiveness would probably decrease because stimulation might be even less.

In a double-blind study by Wood et al,^23^ 74 patients with stages II and III chronic decubitus ulcers in 4 centers, were randomly allocated in a treatment group (*n* = 43) and a placebo (sham treatment) group (*n* = 31), which received standard treatment. Treatment composed of electrical stimulation using low-intensity pulsed direct current (LIPDC) of 300 to 600 μA. After 8 weeks of treatment, 58% of ulcers in the treatment group had healed, whereas in the placebo group only 1 healed, and in the rest of the ulcers, ulcer area increased. A statistically significant accelerated rate of healing (*P* < .0001) was observed.

Reversal of polarity of pulsed direct current during the healing period has been studied. Junger et al^23^ investigated the effect of LIPDC on venous leg ulcers of 15 patients who had not responded to standard compression treatment over 79 months. An intensity of 630 μA was selected initially (frequency: 128 pulses per second; pulse duration: 140 μs) with the cathode placed on the wound for 7 to 14 days. The following 3 to 10 days, the positive electrode was positioned on the wound, and after that specific time frame polarity was reversed again. As soon as significant healing had occurred, intensity was reduced to 315μA (64 pulses per second). Treatment was performed on a daily basis, each session lasting 30 minutes. Mean ulcer area was reduced to 63% (*P* < .01). Furthermore, capillary density was increased to 43.5% (*P* < .039), and improvement of skin perfusion was observed (PtCo_2_ = 13.5 increased to 24.7 to 40 mm Hg being normal) (Table 2).

## DISCUSSION

Current research indicates that LIDC within the range of 200 to 800 μA is effective in promoting and accelerating wound healing. It is emphasized that in no study was blood or serous exudate observed, an indication that the intensity range of 200 to 800 μA is appropriate for low-intensity electrical stimulation. In Table 3, a protocol of application of LIC is presented on the basis of protocols used in studies.

Regarding LIPDCs, studies showed that an intensity of 630 μA is capable of stimulating healing of ulcers that were unsuccessfully treated with standard compression treatment and a current intensity of 300 to 600 μA, for stages II and III pressure ulcers. Thus, an intensity range of 300 to 630 μA appears to be an intensity of choice for treating these specific wounds.

The intensity proposed ranges from 300 to 630 μA on a daily basis for at least 30 minutes for 4 to 8 weeks. Reversal of polarity may be applied, and frequencies of 130 Hz may also be applied. Reversal of polarity in LIPDC has been proposed on the 3rd to 10th day of treatment, provided that no infection has taken place. Reversal may be repeated whenever wound healing has reached a plateau.

Another recommendation can be regarding wounds that have failed to heal using other forms of electric stimulation. The selection of the reverse polarity to the 1 used previously is proposed as employed in the studies by Wolcott et al,^18^ Carley and Wainapel,^22^ and Junger et al.^23^ The protocols presented in Tables 3 and 4 are then suggested.

A comparison of the results of studies on LIDC and LIPDC reveals that their results, despite the numerous differences in protocols, populations studied, and outcome measures, are largely comparable, a fact that weakens the initial hypothesis in the “Results” section, that pulsed LIC might be less effective in wound healing than LIDC.

Intensities of 0.001 to 200 μA and 800 to 1000 μA have not been studied, in either continuous-direct or pulsed-direct LIC. It can only be postulated that intensities of 800 to 1000 μA are effective, because amplitudes of 800 μA and 1 mA were both proven to be effective, although in different waveforms (800 μA in direct current and 1000 μA in alternating current).

A general lack of clinical studies demonstrating no effect of MCs on wound healing was observed. Only 1 study by Katelaris et al^25^ found MCs not to be statistically significantly beneficial for wound healing but this study was not included because this result was probably due to the cytotoxic effect of povidone iodine, as reported by Kloth,^10^ which was used in conjunction with stimulation. Therefore, it can be supported that LIC in wound healing appears to be effective.

Regarding methodological issues, retrieving studies using LIC for wound healing was challenging and required rigorous search strategies. This can be attributed to the lack of differentiation of LIC from other currents of an intensity over 1 mA in the literature, commonly referred to as electrical stimulation in general.

It has to be underlined that in all studies the control or sham-treatment group received standard wound care; therefore, treatment was not withheld, which would be contrary to basic medical ethics. Thus, the control group was a standard-treatment group, and acceleration of rate of healing was in relation to standard treatment and not to no treatment at all. This fact supports that LIC could not be used alone but could be used in conjunction with standard wound care as current research suggests.

A definite conclusion and generalization could not be reached regarding the effectiveness of LIC on wound healing. Only regarding intensity, is there an agreement among studies. All other parameters vary across trials. The effectiveness on a specific type of ulcer could not be established because of the small number of studies for each type of wound. The LIC generators used in the studies have been discontinued, a fact that is of limited significance because parameters and technical characteristics are adequately presented in all studies. Furthermore, another point to be taken is the presence of, to a certain extent, varying outcome measures and criteria, which have been used in studies, a fact that impedes comparison of results and reaching conclusions. Still, the positive results indicate that LICs appear to have a beneficial effect on stimulation and rate of wound healing. The factors mentioned above prevent conclusions on the efficacy and extent of efficacy of LICs in stimulating and accelerating wound healing.

The *clinical implications* of this study may also be considered. Wound healing is a challenge and a delicate healthcare issue for the clinician. Physicians, nurses, physiotherapists, and other members of the rehabilitation team occasionally have to dedicate treatment time on wound care.^26^ Healing is sometimes delayed, and the wound may not respond to standard treatment. These constitute implications, which require a part of patient services to be focused on wound healing. As a result, other healthcare issues might be overlooked or receive less attention, or the presence of the wound itself might slow down rehabilitation progress, impede patient recuperation, and discharge from hospital. Overcoming or restricting the effects of lengthy or treatment-resistant wound healing may enable the healthcare professional to address other health issues such as training transfers to a tetraplegic patient with a pressure sore in the sacral area. Furthermore, hospitalization may be reduced reflecting faster rehabilitation of the patient, improvement of patient services, and reduction of cost of care.

The review may also underline the need for a multidisciplinary approach to wound care, through exploring and gathering evidence on the effectiveness of LIC stimulation, a treatment applied by physiotherapists and physicians, who are a part of the rehabilitation team, as well as the nurse and other rehabilitation professionals.

Research studies unanimously support the efficacy of LIC, still the number of studies on the topic is limited and further research is needed to establish the effectiveness of LIC on promoting and accelerating wound healing. Future research may focus on specific wound types such as diabetic ulcers, or alternative methods of application, for instance, implanted electrodes. The type of electrical current used could be specified to direct research toward establishing the most effective treatment parameters and forms of current.

## CONCLUSION

The evidence available indicates that LIC appear to accelerate wound healing. Regarding the selection of intensity, LIDC (continuous or pulsed) appears to be effective in the range of 200 to 800 μA, and polarity may or may not be reversed. Further research is required to elucidate the effect of LIC on wound healing.

## Figures and Tables

**Table 1:** Low-intensity direct current randomized-controlled trials studies597*

Low-intensity direct current RCT studies	Wolcot et al^18^	Carley & Wainapel^22^
Sample	*n* = 83	*n* = 30
Type of wound	Ischemic wounds	Ulcers over sacrum or lower limb (below knee)
Groups	Treatment group (1 group). Eight patients presented with bilateral wounds. One side was treated with LIDC (*n* = 8) and the other received standard care (*n* = 8)	Electrical stimulation treatment along with conventional treatment group (*n* = 15) or conventional treatment group (*n* = 15)
Treatment	Intensity: 200–800 μA. Three sessions/d, 2 h per session. Polarity was reversed provided that no infection was present on day 3. Treatment was continued to full wound healing	Intensity: 200–800 μA for 2 h, twice daily, 2- to 4-h interval, 5 d/wk, for 5 weeks. Day 3: polarity was reversed unless infection appeared. Polarity was reversed on plateaus
Electrode placement	Negative electrode was placed on the wound and the positive electrode proximally	Negative electrode placed on wound and positive electrode proximally
Results	45% of wounds healed completely (mean 9.6 weeks). The rest reached partial healing up to 64.7% over 7.2 weeks	Wound healing was accelerated 1.5–2.5 times in the LIC group compared with the conventional treatment group, and less debridement, less discomfort, and resilient healed scars were observed
	Bilateral ulcer group: 6 of 8 LIDC-treated ulcers completely healed, 2 of 8 healed up to 70%. Other side: 3 of 8 ulcers did not heal, 3 of 8 healed less than 50%, and 2 of 8 healed up to 75%.	

* RCT indicates randomized-controlled trials; LIDC, low-intensity direct current.

**Table 2:** Low-intensity pulsed direct current randomized-controlled trials studies

Low-intensity pulsed direct current RCT* studies	Wood et al^23^	Junger et al^23^
Sample	*n* = 74 patients in 4 centers	*n* = 15 nonresponsive to standard compression treatment over 79 mo
Type of wound	Stages II and III, chronic decubitus ulcers	Venous leg ulcers
Groups	Treatment group (*n* = 43) and placebo (sham treatment) group (*n* = 31) standard treatment	Treatment group
Treatment	Low-intensity pulsed direct current of 300–600 μA, daily, for 8 wk	Treatment: 38 days daily, session duration, 30 min. Intensity 630 μA (128 pps, pulse duration—140 μs). On significant healing, intensity was diminished to 315 μA (64 pps).
Electrode placement	Not specified	Cathode electrode on wound for 7–14 d. Following 3–10 days, positive electrode positioned on wound, then polarity was reversed again.
Results	8 wk—accelerated rate of healing (*P* < .0001). Ulcers healed in treatment group—58%, 1 healed in placebo group, in remaining ulcers, ulcer area increased.	In 13/15 mean ulcer area, 63% (*P* < .01) (reduced)
		2 ulcers healed completely, capillary density 43.5% (*P* < .039) (increase).

* RCT indicates randomized-controlled trials.

**Table 3:** Low-intensity direct current proposed parameters on the basis of protocols used in studies (presented in Table

Intensity	200–800 μA (negative electrode on wound)
Treatment time	2 h
Times/d	2 to 3 sessions with a 2- to 4-h interval
Times/wk	5 d/wk
Duration of treatment	5 to 9 wk

**Table 4:** Low-intensity pulsed direct current proposed parameters on the basis of protocols used in studies (presented in Table

Intensity	300 to 630 μA (negative electrode on wound, stable polarity or reversal of polarity on 3 to 10 days or when on plateau)
Treatment time or times/wk	30 minutes minimum per day
Frequency	130 Hz
Duration of treatment	4 to 8 wk
